# How a sample of English stop smoking services and vape shops adapted during the early COVID-19 pandemic: a mixed-methods cross-sectional survey

**DOI:** 10.1186/s12954-021-00541-0

**Published:** 2021-08-31

**Authors:** Sharon Cox, Emma Ward, Louise Ross, Caitlin Notley

**Affiliations:** 1 Department of Behavioural Science and Health, University College London, London, UK; 2 Norwich Medical School, University of East Anglia, Norwich, UK; 3 National Centre for Smoking Cessation Training, Dorchester, UK

**Keywords:** Vape shops, COVID-19, Pandemic, Smoking cessation, Stop smoking services

## Abstract

**Background:**

The COVID-19 pandemic in England led to major changes in the delivery of support via stop smoking services (SSS) and to the widespread temporary closure of bricks and mortar e-cigarette retailers (vape shops herein). The impact of disruptions across the smoking cessation support landscape has not been fully documented. The purpose of this study was to capture how SSS and vape shops in England were affected and adapted their ‘business as usual’ during the early months of the COVID-19 pandemic.

**Method:**

An online cross-sectional survey was conducted between March and July 2020. Surveys were disseminated through online networks, professional forums and contacts. Open-ended qualitative responses were coded using thematic analysis.

**Results:**

Responses from 46 SSS and 59 vape shops were included. SSS were able to adapt during this period, e.g. offering a remote service. A high percentage (74.6%) of vape shops had to close and were unable to make changes; 71.2% reported business declining. For both vape shops and SSS qualitative data revealed practical challenges to adapting, but also new pathways to support and co-working.

**Conclusion:**

The closure of vape shops appears to have most impacted smaller bricks and mortar shops affecting businesses by decline in customers and impacting staff (furlough). For those services that could stay open there may be lessons learned in how to support vulnerable and disadvantaged people who smoke by considering new pathways to support.

## Introduction

It is estimated since the start of the pandemic that over one million people who smoke have made a quit attempt (an estimated additional 440,000, compared to pre-pandemic levels) in Great Britain [1]. However, the English NHS Stop Smoking Services (SSS), which offer the ‘gold standard’ treatment of combined behavioural and pharmacotherapy support [2], faced the unusual challenge of how to respond to increased demand for support during social distancing measures. At the same time, e-cigarettes were not included in the government’s definition of essential items, and e-cigarette retailers (vape shops herein) had to close [3]. E-cigarettes are now the most common choice for people who smoke when making a quit attempt in England [4], and there is growing evidence for their efficacy for cessation in trials [5]. For context, people could still purchase tobacco and e-cigarettes and liquids (often tobacco industry manufactured) from convenience stores and supermarkets. Although many vape shops have established online markets which were able to stay open, many smaller, locally based ‘bricks and mortar’ shops did not have this option. Many people engaging in a quit attempt during the COVID-19 pandemic might opt for an e-cigarette and/or attempt to quit with help from the SSS. This study aimed to explore how SSS and vape shops in England adapted during the early pandemic period (March 2020–July 2020), a time of national travel restrictions when the advice to all was to stay at home except for essential reasons.

A recently published survey of local authorities in Great Britain by Action on Smoking and Health (ASH) [6] shows that at the time of their survey (August–September 2020, 5 months after the first national lockdown was announced), just 18% of SSS were offering face-to-face support but this was supplemented by 98% offering telephone consultations and 60% offering online video support. The report highlights that the flexibility of this support was welcomed by patients. Furthermore, the report highlights that the majority of services (59%) adapted service delivery for those patients recorded as vulnerable.

Many adults with pre-existing and severe health conditions were advised by the government to shield or to isolate with minimal contact with others outside their homes [7]; this would have presented both SSS and vape shops aiming to support these people who smoke with unique and unprecedented challenges. As smoking is more frequently observed in socially disadvantaged and clinically vulnerable populations [8, 9], including those at greater risk of severe illness because of respiratory health comorbidities, e.g. chronic obstructive pulmonary disease (COPD), it is important to understand how this was managed and what special adaptations were made. If special adaptions were not made, then key groups may have been excluded from support, and opportunities to engage with important populations missed. If special adaptations were made this could be useful for identifying and developing new ways of working.

This study aims to complement the findings of the ASH survey [6]. Further in the present study, findings from SSS are triangulated by including a snapshot of how e-cigarette retailers were also affected during early lockdown. The aims of this study were to (1) survey how those working within front-line NHS and local authority commissioned SSS and vape shops adapted during the early COVID-19 pandemic, (2) to document what changes were made to usual practice, and (3) to document how the needs of vulnerable people who smoke, defined as those within the shielded list or with health and social needs that make them vulnerable to COVID-19, were being met during this period and to identify potential new ways of delivering services.

## Methods

### Design and setting

A cross-sectional online survey in England conducted between March and July 2020.

### Registration

This study was preregistered on the Open Science Framework (https://osf.io/b3xcy/ [10]).

### Ethical approval

Ethical approval was received from University of East Anglia REF: 2019/20–133; participants were informed their data would remain anonymous and they could withdraw from the study at any time during the survey without penalty.

### Participants

Fifty-two individual responses from SSS and 70 vape shops started the survey; after discounting incomplete responses (with > 5% of missing data [11]), complete data from 46 SSS and 59 vape shops were included. Table 1 presents the percentage of services across English regions.

**Table 1:** Descriptive characteristic and survey response

	Stop smoking service (SSS) n = 46 (%)	Vape shops n = 59 (%)
Region		
South England	22 (47.9)	32 (54.2)
North of England	18 (39.1)	14 (23.8)
Midlands	6 (13)	13 (22)
Able to stay open in some capacity?		
Yes, but with changes	35 (76.1)	13 (22)
Yes, no changes	9 (19.6)	2 (3.4)
No	2 (4.3)	44 (74.6)
Did your service/business furlough any staff?		
No	43 (93.5)	12 (20.3)
Yes	3 (6.5)	47 (79.7)
Did your service/business make any changes to business as usual?		
No	–	3 (5.1)
Yes	46 (100)	56 (94.9)
*Vape shops only*: What best describes the health of your business?		
Business has declined		42 (71.2)
Business is doing better		4 (6.8)
Business is more or less the same		3 (5.1)
Part or all of my business is at risk of closure		3 (5.1)
Not answered		7 (11.8)
Special arrangements in place for vulnerable people who smoke?		
No	33 (71.7)	17 (28.8)
Yes	13 (28.3)	21 (35.6)
Unsure	–	9 (15.3)
Not answered	–	12 (20.3)
Started to work with other organisations?		
No	39 (84.8)	57 (96.6)
Yes	7 (15.2)	2 (3.4)
Cost involved in these extra measures?^+^ (for those who stayed open)		
No	3 (6.8)	12 (57.1)
Yes	15 (34.1)	9 (42.9)
Unsure (unable to answer) or not applicable	26 (59)	–
Considering implementing these new changes in the longer term?*		
No	6 (20)	12 (60)
Yes	24 (80)	8 (40)

^+^SSS n = 44, Vape shops = 21**:** *SSS n = 30, Vape shops n = 20. Furlough refers to the UK government COVID-19 job retention scheme, allowing employers to suspend employment in the absence of work with a government salary subsidy

### Procedure and measures

Both surveys asked about the nature of the support given to people who smoke, changes to service delivery as a result of the pandemic, barriers and facilitators to ongoing support, and plans for future service delivery. Surveys are published online and available at (https://osf.io/b3xcy: [10]). Recruitment was advertised at no cost by SC and CN online (Twitter/Facebook). We sought responses from service leads and frontline staff. The survey for SSS was also sent out by email to tobacco control and policy stakeholder personal contacts and disseminated through smoking cessation service networks, via the National Centre for Smoking Cessation and Training. The vape shop survey was distributed through a list of English vape retailer contacts; this list was developed by researchers working with authors (EW, CN) and includes all retailers registered with the Independent British Vape Trade Association (IBVTA) and this was supplemented by web searches of retailers not registered with the IBVTA. Upon seeing the study advertised via social media, The Planet of the Vapes, a website for vape consumers and businesses, also advertised the survey. Participation was voluntary and no incentives were offered.

The surveys were run online using Qualtrics XM software. Once participants had consented, they were asked to complete the survey. We asked for only one response per business/service to avoid duplication. Participants were asked only to complete the survey if they had full knowledge of how the pandemic had impacted their service. The survey was a mix of both multiple choice and open text items. Open text responses allowed for people to explain in greater detail the processes and changes they had made. Upon completion participants were thanked for their responses and debriefed.

### Analyses

Quantitative data are presented as exploratory descriptive statistics only. There were no planned comparisons. Sample size (*n*) and percentage (%) are reported for categorical variables and means and standard deviations (SD) for continuous ones. The open text questions were analysed using a combined deductive (to meet research aims) and inductive thematic analysis (to allow novel themes to emerge). Analysis was led by CN [12], with verification of coding and further analysis by EW until consensus of reported themes was reached.

## Results

Forty-six individual responses were received from SSS and 59 vape shops participated. We were unable to determine response rate as recruitment was voluntary through extended networks and online promotion.

Table 1 presents the quantitative data from the survey. There are several key findings in relation to our primary aim.

### Staying open in some capacity and adapting the ‘business as usual’ service

The results show that 95.7% of SSS stayed open in some capacity, even if only offering initially a telephone-based remote service. Only 6.5% of SSS furloughed any staff (furlough refers to COVID-19 job retention scheme offered by the UK governments; it enables employers to suspend employment in the absence of work with a government salary subsidy); this is likely to reflect the ability of SSS to be able to continue with the support of the local authorities. However, all surveyed SSS made changes to service delivery.

**Fig. 1:**
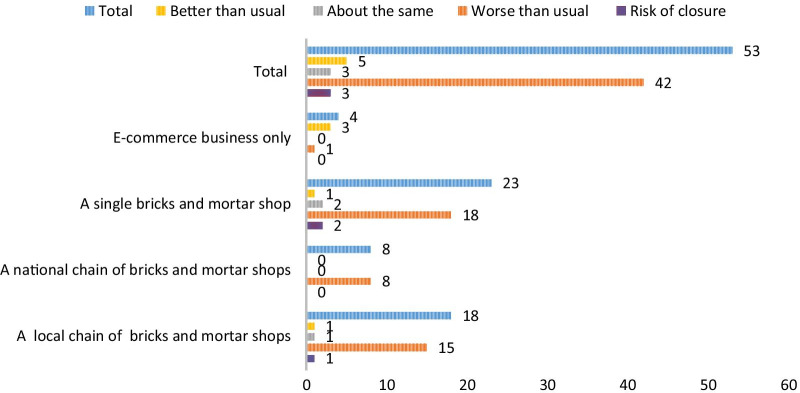
Response to current health of business by type of e-cigarette retailer

**Fig. 2:**
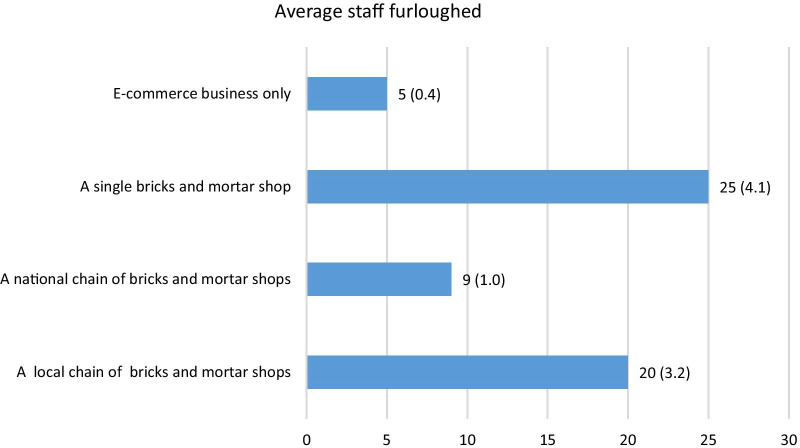
Average number of staff furloughed by type of e-cigarette retailer. Mean number of staff furloughed and standard deviation shown in parenthesis

The majority (94.9%) of vape shops told us that they were affected by the lockdown, see Table 1, with 71.2% reporting business had declined. (Figure 1 shows how business was affected by type of vape business.) Of the vape shops that were able to stay open ‘in some capacity’, these were online retailers (though one said they could not) and business was the same or doing better. Of those that closed 77.3% were bricks and mortar vape shops, either independent, part of a local chain of stores, or 20.5% were part of national chain. A large percentage (79.7%) of vape shops furloughed staff; Fig. 2 presents the average number of staff furloughed by vape business type, as can be seen, smaller single vape shops reported business being worse than usual and furloughing a higher number of staff.

Data show 18.3% and 35.6% of SSS and vape shops, respectively, reported adapting their service for the needs of people who smoke who were deemed especially vulnerable (Table 1). Only 15.2% and 3.4% of SSS and vape shops reported working with other agencies or organisations. Both SSS and vape shops report an extra cost involved in implementing new changes.

### Qualitative findings

Tables 2 and 3 report the open ended responses from vape shops and SSS, respectively.

**Table 2:** Open ended responses—qualitative coding—vape shops

Vape shops	Theme/s	Example
What arrangements did you put in place for vulnerable people who smoke?	Distancing measures in shop PPE Increased information for customers, e.g. online/telephone consultations Safe remote delivery Telephone support Longer remote contact hours Increased online range Partnership with social care Safe collection from shop Extra costs for: webhosting/paypal PPE/cleaning products Petrol/car insurance for delivery Postage Charitable donations for social care partnership Low value delivery Some had no extra costs as systems already in place	“We made contact-free daily home deliveries and sanitised products before sealing them in packages. We also provided (and always have) text, telephone, and Facebook support whenever needed. We also provided free of charge, hand sanitiser if requested, and brought food supplies to those who could not get items themselves”
We are trying to identify new pathways to good practice, please tell us anything that has worked for you	Same day home delivery Local pick up service Zoom webinar for smoking cessation counsellors/practitioners to demystify e-cigarettes Click and collect Keeping upbeat for shielders Facebook messenger to exchange photographs so could advise on device consumables Extra care and attention paid to regular shielding customers The above expanding on the arrangements above No testers (negative) Limits to numbers in shop	“We have stopped offering testers, as even with the hygiene tips they are handheld devices and could increase risk of a virus spreading”
Have you had any feedback from customers about the service change (please briefly outline)	Customer satisfaction (both informally and google reviews, the latter good for business) Smoking relapse prevention *but also* Smoking relapse Business growth Delays to delivery Staff and customers found it hard to get to grips with new online working	“Customers appreciated the same day delivery but could not understand why we were not classed as an essential service” “Some customers who used the Local Pickup Service have told us that it stopped them from buying cigarettes” “Absolutely. We have had countless 5-star Google and Facebook reviews during and after lockdown, all of which were positive. It has helped our business grow and has motivated the staff to continue to provide the best service possible to all our customers” “Many of our regular customers went back to smoking due to the easier availability of cigarettes when shops were closed and online delivery was chaotic across the country. Devices don't sell much online as people need the advice and personal consultation”
Are you considering implementing any of changes that you made during COVID-19 in the longer term? Yes. Please tell us: Text	Maintain e-commerce Keep local pickup option Keep local delivery service Coordinated efforts Shop closure	“Ongoing efforts to strive to work better with smoking cessation stakeholders” “We are planning on going fully online with no shop front due to recession fears and a second spike”

**Table 3:** Open ended responses—qualitative coding—stop smoking services (SSS)

SSS	Theme/s	Example
What arrangements did you put in place for vulnerable people who smoker?	SMS support Longer prescriptions Posting prescriptions/prescription collection Telephone consultations App Partnerships with vape industry (e.g. wholesalers, vape shops) Collaborations with drug/mental health services Costs: Postal/mileage delivery costs App licence costs Extra IT equipment for staff BUT savings on venue and staff millage	“For those who were self-isolating we either arranged for their pharmacy to post products to them or we advised the client to join the 'Good Sam' app and a volunteer would pick up their products We also arranged for those who were self-isolating but who wanted Champix for the appendix 1 of the PGD to be completed by the pharmacist over the phone Two vouchers which is equal to a month’s supply of product were posted to clients so they did not have to go to pharmacies as many times and stand in long queues”
We are trying to identify new pathways to good practice, please tell us anything that has worked for you	Telephone consultations Welfare checks and opportunistic smoking cessation Joint working with vape shop Peer group support on teams Staff meetings/training on zoom Webinar training sessions for other HCPs Alternative to CO testing Clients missing less appointments/completing more paperwork Social media recruitment successful Redeployment to welfare roles provided new opportunity to reach clients	“We have the same quit conversion rate at 4 weeks as face to face consultations. A key factor is possibly client's do not miss many appointments, making it more likely they will quit even without the perceived benefit of face to face Clients get a quit guide and top tips in the post when they set a quit date, this was hit and miss before and dependant on the advisor. Similarly, at the 12-week quit point they get a certificate, staying quit guide, evaluation form and sae which before was very hit and miss. We are now receiving a beneficial amount of evaluation forms back About half of our small team were redeployed to make welfare checks and were involved in emergency food parcel deliveries. We used the opportunity when speaking to people seeking stop smoking support to check they had access to food and basic necessities and referred them for emergency help if necessary No CO monitoring so we have used a breathing challenge identifying how long they can breathe in and out and hold and working to improving their lung capacity”
What has been difficult about delivering the service remotely?	No CO monitoring Dealing with client emotional issues Impact on rapport Demands from commissioners Difficulty engaging pregnant women IT issues Demands on resources, e.g. more demand and less staff, missing calls, constant calls, long calls, IT issues working from home, confidentiality issues working from home	“Advisors missed the relationship and face to face interaction. No Co monitoring as no face to face so had to take clients word about staying quit and motivation of seeing CO reading go down to non-smoker used to be a good talking point and motivation for clients” “The pregnancy side has proved more difficult, more women declining the midwives, possibly due to no co screening or maybe it is just easier to say no via phone. It could be a training need for midwives”
Have you had any feedback from customers about the service change (please briefly outline)	More convenient than face to face Checking in with shielding clients Appreciation of service adapting to remote contact Increased rapport	“Being in lockdown has helped some people to avoid other smokers” The regular contact has been valuable to them and often a comfort that someone is looking out for them” “Patients are so grateful that we care about them during the pandemic and I personally have learnt so much more about them their lives and interests than I normally would of”
Are you considering implementing any of changes that you made during COVID-19 in the longer term? Yes. Please tell us: Text	More virtual clinics Implementation of smokefree app Continue telephone support	

### Practical arrangements

Vape shops (that were able to react) and SSS responded to the immediate pandemic crisis by implementing practical changes to service delivery, for example, offering remote telephone consultations for cessation support, and avoiding personal contact by offering ‘click and collect’ purchasing services for vape supplies via telephone as well as online orders:

We made contact-free daily home deliveries and sanitised products before sealing them in packages. We also provided (and always have) text, telephone and Facebook support whenever needed. We also provided free of charge, hand sanitiser if requested, and brought food supplies to those who could not get items themselves. (Vape shop)

SSS responded quickly by supplying longer than usual prescriptions of stop smoking medication, particularly for vulnerable clients and those self-isolating. Deliveries of stop-smoking products were also arranged by some services, and similarly some vape shops offered a delivery service to customers who were self-isolating or shielding—however, noting that this arrangement created extra financial costs for either the customer (if passed on) or the business.

### New pathways

Both SSS and vape shops adopted some innovative practices in response to the pandemic. SSS offered video conferencing meetings and appointments, and some even attempted group support delivered using video conferencing. A major service change brought about as a result of infection control measures was the abrupt stopping of all carbon monoxide (CO) monitoring. In response to this, SSS developed innovative ways of checking in with existing clients to establish smoking status:

No CO monitoring so we have used a breathing challenge identifying how long they can breathe in and out and hold and working to improving their lung capacity (SSS).

Some SSS staff were redeployed to deliver food parcels and medication within the community and took advantage of being able to make ad hoc ‘welfare checks’ to make contact with people who might be isolated. These ad hoc checks sometimes reportedly resulted in quit attempts that may not otherwise have been planned. Some SSS also described new pathways for joint working with vape shops to offer remote support for clients attempting to quit and stay quit from smoking. Equally, vape shops also described working with SSS, with some organising remote online seminars to explain products to SSS staff. Taking an innovative approach, some vape shops described using Facebook messenger or WhatsApp to send photographs of devices to customers to explain processes such as how to use devices and how to change components.

### Feedback from clients/customers

When asked about feedback from clients, both SSS and vape shops overwhelmingly reported that they had had positive feedback. Customers reportedly understood the difficult times and the need for shop closures or remote support offers. They were hugely appreciative that services were able to continue to support them, and in the case of shops, to supply e-cigarette consumables, despite the challenges. Some customers actually preferred remote support, finding it convenient not having to travel to appointments or to make purchases. Others were extremely grateful for service continuation while they were having to self-isolate or shield:

Patients are so grateful that we care about them during the pandemic, and I personally have learnt so much more about them, their lives and interests than I normally would… (SSS)

Vape shops commented that remote provision was critical for enabling clients to remain smokefree, but also drew attention to the mismatch between tobaccos being available to purchase through ‘essential’ shops that remained open. Vape supplies were harder to purchase due to shop closures, as vape shops were deemed ‘non-essential’. There was real concern that clients may have relapsed to smoking as a direct result of this anomaly:

Many of our regular customers went back to smoking due to the easier availability of cigarettes when shops were closed and online delivery was chaotic across the country. Devices don't sell much online as people need the advice and personal consultation. (Vape shop)

### Implementation of long-term changes

SSS and vape shops talked positively about changes that had been implemented that would be continued in the long term, including remote support provision, click and collect and online purchasing options, and the use of video conferencing for staff training, meetings and client support. Positively, there was also discussion of continued joint working between vape shops and SSS:

Ongoing efforts to strive to work better with smoking cessation stakeholders. (SSS)

It was noted that there was an increased desire and willingness to promote digital support options for behaviour change, such as apps.

## Discussion

The overall landscape of smoking cessation support changed substantially during the early phases of the COVID-19 pandemic. SSS quickly switched to remote service provision, and many vape shops without an online market were forced to close completely, but some were able to adapt by offering click and collect or delivery services for vape supplies. Overall, vape shops were more negatively impacted than SSS as evidenced by reports of business being worse than usual and furloughing staff. E-cigarettes not being deemed essential products appears to have affected small independent shops in particular, who report struggling to adapt. Simultaneously, evidence suggests that there was a surge of interest in smoking cessation [1], unfortunately implying that as more of the population were attempting to quit smoking, the support available to them both through NHS routes and less formal but popular routes, such as vape shops, was diminished.

There were many positive reported examples of good practice—SSS staff were able to offer remote appointments and engage in online training. Vape shops, particularly those connected to larger chains with a more secure infrastructure, were able to offer remote delivery options and also were able to use video conferencing software to explain products to customers; this is similar to what was reported in a report of SSS by ASH [6]. For SSS staff, there were redeployments to other areas of public health need, but our data positively demonstrate examples of how this enabled them, with their training, to identify people willing to quit and to promote smoking cessation at given opportunities.

Also positively, it was evident that both SSS and vape shops made particular efforts to meet the needs of vulnerable people who smoke. Delivery options were offered to the clinically vulnerable or those having to self-isolate. Many of the extra measures came with an additional cost, with smaller vape shops reporting having to pass this on to customers with ‘low value’ orders. E-cigarette use has increased in all > 1 year people who smoke in England, however analysis by Kock et al. [13] shows that use is highest amongst the most disadvantaged social grades (e.g. those working within routine and manual trades). Thus, speculatively, the burden of taking on extra costs may not be evenly distributed across all social gradients. Furthermore, small independent vape shops are often located in the most deprived communities [14]. This suggests that a valuable community smoking cessation asset may have been lost to some populations most at risk of continued smoking, and most susceptible to the worst impacts of the COVID-19 pandemic. It is also a distinct possibility that recent quitters may have relapsed to tobacco smoking, as a more accessible way of using nicotine during lockdown than vaping. Indeed, research is now needed on how vapers accessed their products and the association between vape product availability and vaping status.

The unintended consequence of compulsory vape shop closures is an example of how sweeping population measures can have grave impacts for already disadvantaged communities, and how pandemic policy measures may serve to widen health inequalities. The impact of smoking relapse across the social gradient warrants future attention, this may help to direct resources and tailor interventions. Given the Department of Health and Social Care’s aim to be ‘smokefree’ by 2030 [15], defined as a smoking prevalence rate of less than 5%, it is important to ensure policies and resources are now targeted at those groups with high smoking prevalence rates to remedy any interruption to the pace of this change. Researchers can assist with this goal by starting to highlight those groups who have reduced uptake in smoking cessation support over the pandemic period, and as mentioned above, those who have shower higher pandemic period rates of relapse.

### Limitations

This study was limited by the brief self-report cross-sectional nature of the data and the number of respondents was small. There may have been misunderstanding of terminology, e.g. vulnerable, although definitions were provided. The survey was targeted at SSS and vape shops across England and offers a ‘snapshot’, but the sample are self-selecting as only those motivated to complete the survey would have replied. The list of vape suppliers was comprehensive but those who responded may not be representative of the sectors wider experience. It may be the case the survey responses highlight particularly good practice or negative consequences and may miss the ‘standard response’ that may not have been deemed worthy of reporting back via a survey. Similarly, the qualitative data are illuminating and informative, but descriptive and limited by possible selection bias. Clearly there is a need to monitor smoking cessation service delivery, both through formal commissioned routes, and less formal community assets, as the pandemic continues. There is also a need to triangulate the self-report and qualitative data reported here with larger epidemiological data as it becomes available, and with both tobacco and e-cigarette sales data over the time of the pandemic.

## Conclusion

The landscape of smoking cessation support has changed and adapted during the COVID-19 pandemic. There are clear positive innovations that services may wish to continue to implement, such as outreach support, delivery services, and remote support via phone or video calls. However, this study was written and conducted during the pandemic. How the changes to services will have affected people who smoke may not be realised for some time.

## Data Availability

The datasets used and/or analysed during the current study are available from the corresponding author on reasonable request.
